# Indolent Progression of a Persistent Positron Emission Tomography (PET)-Negative Ground-Glass Pulmonary Nodule to Pulmonary Adenocarcinoma Following Nine Years of Radiographic Surveillance: A Case Report

**DOI:** 10.7759/cureus.115300

**Published:** 2026-08-27

**Authors:** Talyn Smith, Chenxi Shi, Saima Memon

**Affiliations:** 1 College of Osteopathic Medicine, Kansas City University, Joplin, USA; 2 Pulmonary and Critical Care Medicine, Mercy Health System, Springfield, USA

**Keywords:** adenocarcinoma, ck7, lung nodule, surveillance imaging, ttf-1

## Abstract

Indolent pulmonary adenocarcinoma spectrum lesions can remain radiographically stable for years and often demonstrate little to no fluorodeoxyglucose (FDG) uptake on positron emission tomography (PET), making diagnosis and management challenging. We report the case of a 74-year-old Caucasian woman with a remote five-year smoking history and a strong family history of lung cancer who was followed for a persistent right upper lobe ground-glass pulmonary nodule. Despite gradual enlargement, PET imaging performed seven years later demonstrated no significant FDG uptake. Serial computed tomography (CT) scans over the following years showed slow progression from a pure ground-glass nodule to a mixed ground-glass and part-solid lesion with an enlarging solid component, raising concern for an indolent adenocarcinoma. Transbronchial needle aspiration demonstrated atypical epithelioid cells, and immunohistochemical staining was positive for thyroid transcription factor-1 (TTF-1) and cytokeratin 7 (CK7) and negative for p40, confirming pulmonary adenocarcinoma. Repeat PET imaging two years later demonstrated mild FDG uptake despite histopathologic evidence of malignancy. This case highlights the prolonged radiographic evolution of a persistent ground-glass pulmonary nodule and demonstrates that limited FDG uptake does not exclude an indolent pulmonary neoplasm. Development or enlargement of a solid component within a persistent ground-glass nodule should prompt careful reassessment even when the metabolic activity remains low.

## Introduction

Lung cancer has remained the leading cause of cancer-related mortality worldwide and continues to represent a major global health burden despite screening and treatment advancements [1,2]. Non-small cell lung cancer (NSCLC) accounts for approximately 85% of all lung cancers, with adenocarcinoma being the most common histologic subtype [3]. These tumors frequently show up in the peripheral lung and are most often first identified as ground-glass or partially solid nodules on computed tomography (CT) imaging.

Persistent subsolid pulmonary nodules, including ground-glass and part-solid nodules, represent a diagnostic challenge because lesions in this category may demonstrate slow growth and remain radiographically indolent for years. The pulmonary adenocarcinoma spectrum ranges from preinvasive lesions like atypical adenomatous hyperplasia and adenocarcinoma in situ (AIS), to minimally invasive adenocarcinoma (MIA) and invasive adenocarcinoma. This emphasizes the importance of utilizing longitudinal radiographic findings with pathologic evaluation [3,4]. The 2011 International Association for the Study of Lung Cancer/American Thoracic Society/European Respiratory Society (IASLC/ATS/ERS) classification system redefined pulmonary adenocarcinoma and determined it as a spectrum ranging from pre-invasive lesions to invasive disease and emphasized the importance of correlation between radiology and pathology [4-6].

Although many persistent ground-glass nodules remain stable over prolonged periods, development or enlargement of a solid component is an important radiographic feature that is associated with the potential for increasing invasiveness [5,7]. This progression may be more clinically significant than changes in overall nodule size alone and can raise the consideration of tissue sampling or definitive treatment. Current clinical guidelines from the National Comprehensive Cancer Network (NCCN) place an emphasis on serial imaging surveillance for indeterminate nodules [8]. Management can be challenging due to the slow growth and the malignant potential of lesions that fall into this category. When these types of lesions demonstrate long-term stability, they may eventually show invasive transformation, which shows the importance of prolonged surveillance and timely diagnosis if any subtle changes occur. 

FDG-PET may provide additional information during the evaluation of pulmonary nodules. However, its sensitivity can be limited in small ground-glass predominant lesions and tumors with relatively low metabolic activity [9-11]. Consequently, limited or absent FDG uptake should not be interpreted alone as evidence of benign lesions when serial CT imaging demonstrates concerning progression. 

This case demonstrates the diagnostic and management challenges that are associated with a long-standing persistent ground-glass pulmonary nodule. The lesion was initially identified as a 6 x 9 mm pure ground-glass nodule and subsequently evolved over nearly nine years into a larger nodule with a soft tissue solid component. Despite the progression, the lesion demonstrated only low-level FDG uptake on serial PET imaging, with an SUVmax of 1.3 and an SUVmax two years later of 1.7. Bronchoscopic biopsy ultimately demonstrated rare highly atypical glands suspicious for adenocarcinoma, although the limited tissue sample did not provide histologic confirmation. This case emphasizes the importance of recognizing the progressive changes within a persistent subsolid nodule and utilizing serial CT findings with PET and pathologic evaluation instead of relying on prolonged stability or limited FDG uptake alone. 

## Case presentation

A Caucasian woman in her mid-70s, with a remote five-year smoking history and a strong family history of lung cancer, was followed for a persistent right upper lobe ground-glass pulmonary nodule that was first identified approximately nine years prior, measuring 6 x 9 mm (Table 1). She was otherwise asymptomatic from a pulmonary standpoint. Her family history was notable for lung cancer in two of her first-degree relatives, as well as melanoma, which increased concern for an underlying malignant process despite her personal history.

**Table 1:** Chronological evolution and evaluation of the right upper lobe pulmonary nodule. Serial imaging demonstrates gradual progression from a pure ground-glass nodule in 2017 to a part-solid lesion with an enlarging solid component, followed by tissue sampling and staging evaluation in 2026. RUL: right upper lobe, FDG: fluorodeoxyglucose, PET: positron emission tomography, CT: computed tomography.

Date	Study	Lesion size	Key findings
2017	Chest CT	6 x 9 mm	Pure ground-glass RUL nodule
2024	Chest CT	17 mm, with a 9-mm solid soft-tissue component	Development of solid component
2024	FDG-PET/CT	7 x 10 mm	SUVmax 1.3, low-level FDG uptake
2026	Chest CT	16 x 22 mm, with a 9-mm soft-tissue component	Progressive mixed ground-glass and soft-tissue lesion; high suspicion for indolent neoplastic process
2026	FDG-PET/CT	13 x 20 mm, with a 5 x 10 mm soft-tissue component	SUVmax 1.7, mild FDG uptake, hypermetabolic right hilar lymph nodes, and no distant metastatic disease

**Figure 1:**
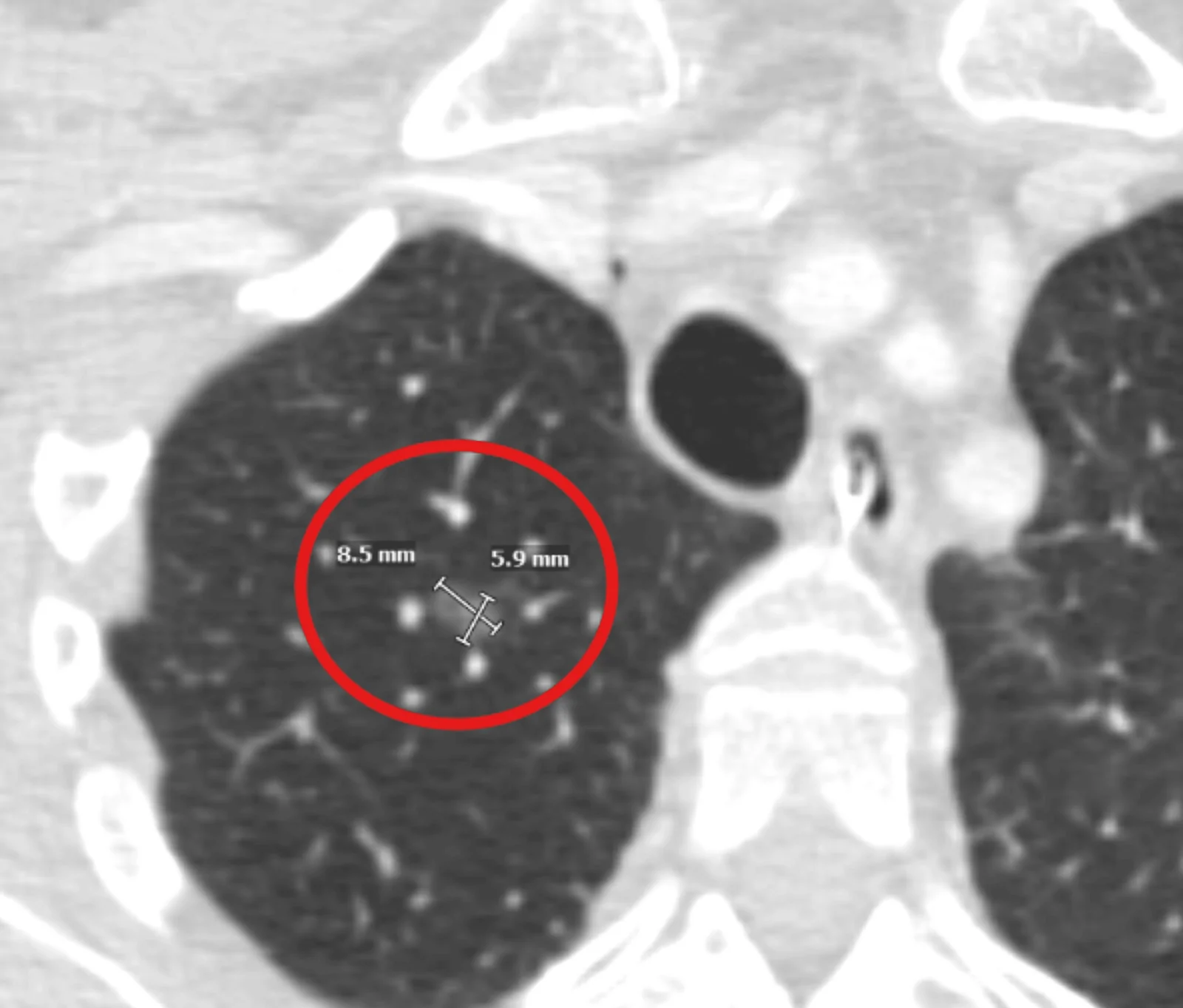
Selected axial contrast-enhanced chest CT image obtained at the time of initial diagnosis, demonstrating a 6 × 9 mm lesion with benign imaging features.

**Figure 2:**
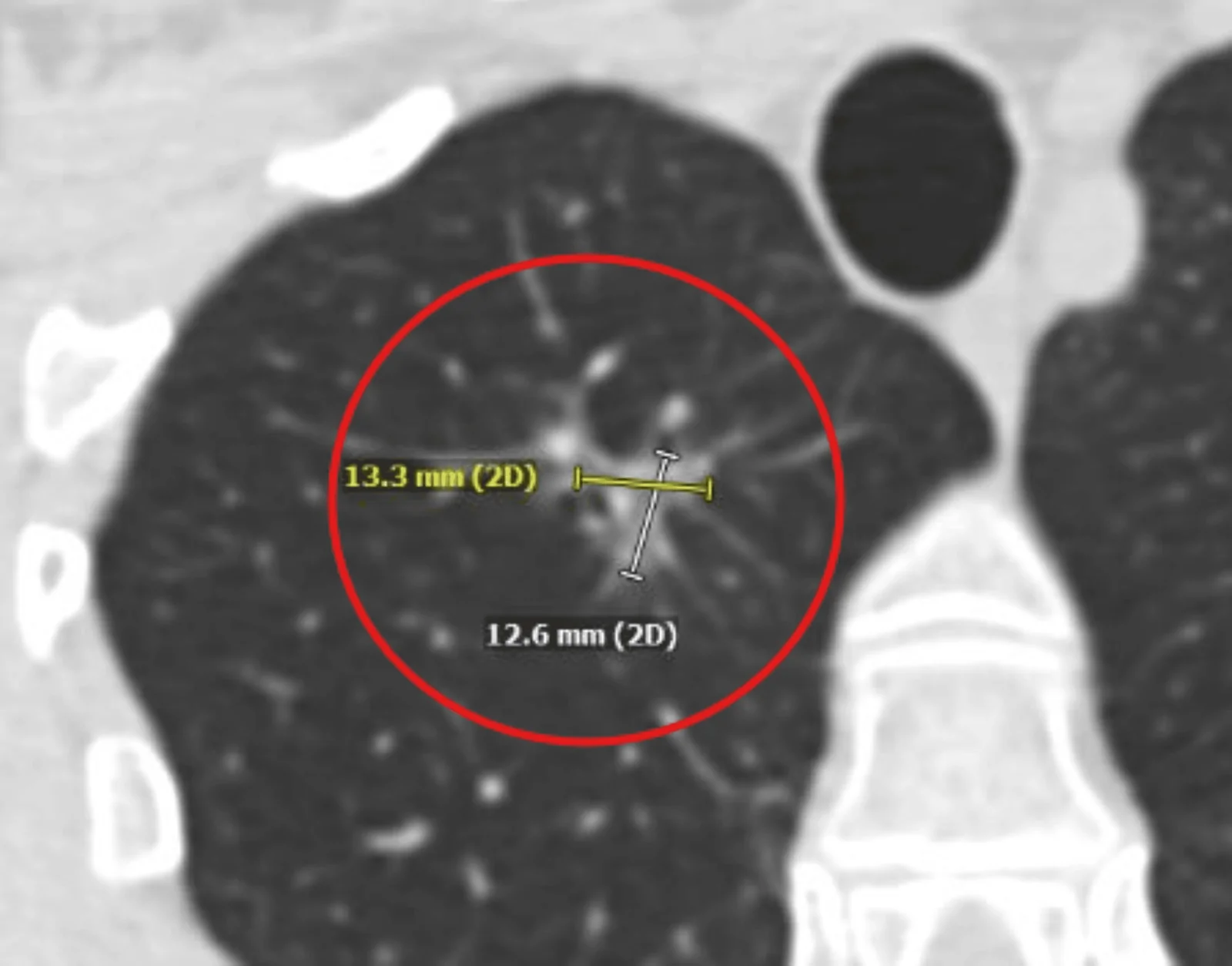
Selected axial non-contrast chest CT image obtained approximately seven years after the initial diagnosis, demonstrating interval enlargement of the right upper lobe subsolid pulmonary nodule with development of an approximately 8-mm solid component.

**Figure 3:**
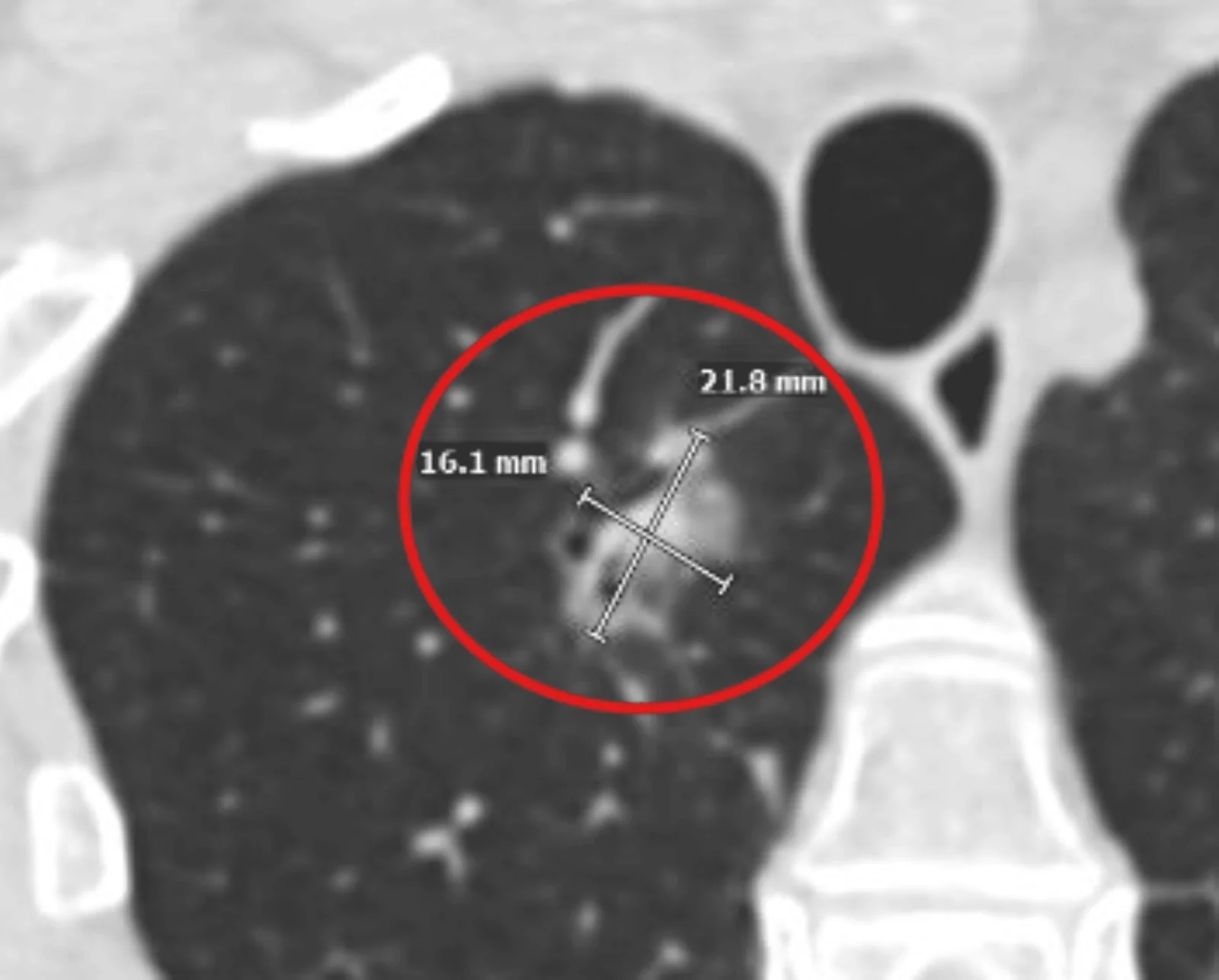
Selected axial non-contrast chest CT image obtained approximately nine years after the initial diagnosis, demonstrating progressive enlargement of the right upper lobe ground-glass nodule, with an increasing solid component compared with prior surveillance imaging.

The right upper lobe pulmonary nodule was initially detected on CT imaging approximately nine years prior, measuring 6 x 9 mm, and characterized by pure ground-glass opacity (Figure 1). Given its small size, ground-glass appearance, and the absence of any concerning radiographic features, conservative management with serial CT surveillance was recommended. Over the next several years, the nodule demonstrated gradual morphologic progression. Follow-up CT imaging performed approximately seven years after the initial diagnosis showed enlargement of the lesion to approximately 17 mm with the development of an 8-mm solid component. PET-CT performed following the development of the solid component demonstrated low-level FDG uptake, with an SUVmax of 1.3. These findings represented a change from the original pure ground-glass appearance (Figure 2). A repeat chest CT performed nine years after the initial finding demonstrated further progression of the mixed ground-glass and soft-tissue lesion measuring approximately 16 × 22 mm, with a 9-mm solid component, both findings that are considered highly suspicious for an indolent adenocarcinoma spectrum neoplasm (Figure 3). Because of the highly suspicious radiographic evidence, soft-tissue sampling and PET-CT were recommended. 

Given the progressive radiographic changes, the patient underwent bronchoscopic transbronchial needle aspiration and biopsy of the right upper lobe lesion. Initial cytology was limited, showing predominantly blood and crushed stromal tissue with a small focus of mildly atypical epithelioid cells (histology performed at an outside facility and image is not available). Biopsy of the nodule demonstrated rare highly atypical glands suspicious for adenocarcinoma in a background of dense fibrosis. The atypical glands were lined by highly atypical cells that were positive for TTF-1 and CK7 and negative for p40. The findings were considered highly suspicious for adenocarcinoma; however, the atypical cells were extremely limited, and additional tissue sampling was recommended for definitive diagnosis and any further studies that might be required.

Mediastinal lymph node sampling was performed during the bronchoscopy. Fine needle aspiration of station 7, 4R, and 4L lymph nodes demonstrated no malignant cells. Station 7 showed polymorphous lymphocytes, histiocytes, and cartilage. Station 4R demonstrated small lymphocyte clusters, mixed inflammation, and rare benign bronchial epithelial cells. Station 4L demonstrated polymorphous lymphocytes, histiocytes, and scant cartilage. 

Following the biopsy, staging evaluation was pursued. FDG-PET/CT demonstrated a right apical ground-glass and soft-tissue nodule measuring 13 x 20 mm, with a 5 x 10 mm soft-tissue component. The lesion demonstrated mild hypermetabolism, with an SUVmax of 1.7. Despite the relatively low level of FDG uptake, the progressive increase in lesion size and development of a large soft-tissue component remained highly suspicious for an indolent neoplastic process. Hyperplastic lymph nodes raised concern for possible nodal metastatic involvement. No additional evidence of metastatic disease was identified. 

The patient's case was reviewed at a multidisciplinary tumor board. Radiation oncology evaluated treatment options, including additional tissue sampling of the lymph node and treatment of the primary right upper lobe lesion with stereotactic radiation therapy at a dose of 55 Gy in five fractions delivered over two weeks. The patient decided to proceed with stereotactic radiation therapy. Molecular testing using a 105-gene Tempus liquid biopsy panel demonstrated microsatellite stability.

## Discussion

Pulmonary adenocarcinoma develops through a well-recognized spectrum of peripheral lung lesions that range from atypical adenomatous hyperplasia to AIS, MIA, and ultimately invasive adenocarcinoma. Lesions found along this spectrum are frequently slow-growing and may remain radiographically stable for prolonged periods, particularly when presenting as ground-glass or partially solid nodules [3,4]. In a recent retrospective study of 322 patients with sub-solid nodules, 19% of patients required treatment within five years, and those with non-solid nodules were less likely to require treatment than part-solid nodules. With the increasing use of low-dose CT screening, persistent subsolid pulmonary nodules are being identified more frequently. Although many lesions remain stable for years, distinguishing the ones that will remain indolent from those that will clinically progress remains challenging [9-11].

This case illustrates several important challenges that come with evaluating a pulmonary nodule, especially a persistent subsolid pulmonary nodule. The patient's right upper lobe lesion was initially identified as a 6 x 9 mm pure ground-glass nodule. Over subsequent years, the lesion developed a large solid component, measuring approximately 17 mm with an 8-mm solid component. Although the lesion had an indolent course of action over many years, it made morphologic progression by development and enlargement of a solid component. Prior longitudinal studies have also shown that persistent subsolid nodules may continue to enlarge or develop suspicious features even after long periods of surveillance [12,13]. The development of a solid component in a ground-glass nodule is particularly important because it can reflect increasing invasive potential [4,7]. In this patient, the change from a purely ground-glass lesion to a part-solid lesion with a measurable solid component represented a more clinically significant change than overall lesion size alone. This emphasizes the importance of evaluating the internal morphology of a persistent subsolid nodule during serial CT surveillance rather than relying only on changes in the diameter. 

This case also highlights the limitations of FDG-PET in the evaluation of indolent pulmonary adenocarcinoma spectrum lesions. The PET that was initially performed after growth demonstrated low-level FDG uptake, with an SUVmax of 1.3, while the repeat PET demonstrated only mild uptake, with an SUVmax of 1.7 despite progressive morphologic changes on CT. Reduced FDG uptake has been shown in AIS, MIA, and predominantly ground-glass lesions because of their relatively low level of metabolic activity [11,12]. Due to this, limited FDG uptake should not be interpreted alone as evidence for malignancy when serial CT scans show progression or enlargement of a solid component. Importantly, this case does not establish that limited FDG uptake directly delayed diagnosis. Instead, it shows the importance of interpreting PET findings in the broader clinical and radiographic context.

Tissue diagnosis can also be a challenging aspect in lesions that have a predominant ground-glass appearance. In this case, the transbronchial biopsy showed rare highly atypical glands suspicious for adenocarcinoma in a background of dense fibrosis. The atypical cells were positive for TTF-1 and CK7 and negative for p40, supporting an epithelial process that is suspicious for pulmonary adenocarcinoma. However, due to the atypical glands being very limited, the pathology report recommended additional tissue sampling for definitive diagnosis and ancillary testing. This is important because the pathology given supports suspicion for adenocarcinoma but does not provide a definitive histologic confirmation of invasive adenocarcinoma. Limited sampling and the potential for sampling error remain important to consider when evaluating persistent subsolid lesions. 

The staging evaluation demonstrated how complex management is in this setting. Mediastinal lymph node sampling from stations 7, 4R, and 4L showed no malignant cells. However, subsequent PET imaging demonstrated hypermetabolic right hilar lymph nodes that were concerning for possible nodal involvement. These findings required the radiographic, pathologic, and clinical information to come together rather than relying on a single diagnostic modality.

Management was discussed in a multidisciplinary tumor board setting. Because of the highly suspicious primary lesion, limited tissue diagnosis, negative sampled lymph nodes, and absence of distant metastatic disease, radiation oncology considered treatment directed at the primary right upper lobe lesion. The patient elected for stereotactic radiation therapy while closely monitoring the lymph nodes. 

Current surveillance guidelines appropriately recommend conservative monitoring of subsolid nodules due to their often indolent behavior [5,6]. However, this case highlights the limitations of relying solely on surveillance when subtle but clinically significant morphologic changes develop over time. In particular, increasing solid component proportion, even in the setting of minimal overall size change, should prompt reconsideration of tissue diagnosis or definitive intervention.

Overall, this case shows three important considerations in the evaluation of persistent subsolid pulmonary nodules. First, prolonged radiographic stability or slow growth does not exclude an adenocarcinoma spectrum neoplasm. Second, development or enlargement of a solid component may be more clinically informative than overall lesion size alone. Third, low-level FDG uptake does not exclude malignancy in ground-glass lesions. Careful assessment, tissue evaluation, staging, and multidisciplinary decision-making are essential in the management of these lesions.

## Conclusions

Persistent pulmonary adenocarcinoma spectrum lesions may demonstrate a very slow radiographic evolution over the course of several years. In this case, a right upper lobe nodule followed for nearly nine years developed into a large part-solid lesion with a progressively enlarging solid component. The lesion demonstrated only low-level FDG uptake despite the morphological changes, emphasizing that limited PET avidity should not be interpreted as evidence of a benign nodule. Development or progression of a solid component within a persistent ground-glass nodule should raise clinical concern for an adenocarcinoma spectrum neoplasm. Long-term surveillance, appropriate tissue evaluation, staging, and management are important when there is evidence of progression, and limited tissue sampling creates clinical concern. 
